# CMR survey in Thalassemia Intermedia patients

**DOI:** 10.1186/1532-429X-16-S1-P267

**Published:** 2014-01-16

**Authors:** Antonella Meloni, Maria Eliana Lai, Stefania Vacquer, Maddalena Lendini, Petra Keilberg, Maria Chiara Resta, Claudio Ascioti, Vincenzo Positano, Massimo Lombardi, Alessia Pepe

**Affiliations:** 1 CMR Unit, Fondazione G.Monasterio CNR-Regione Toscana and Institute of Clinical Physiology, Pisa, Italy; 2 Ospedale microcitemico, Centro Talassemici Adulti, Cagliari, Italy; 3 Centro trasfusionale, Osp. Giovanni Paolo II, Olbia, Italy; 4 Struttura Complessa di Radiologia, OSP. SS. Annunziata ASL Taranto, Taranto, Italy; 5 Struttura Complessa di Cardioradiologia, P.O. "Giovanni Paolo II", Lamezia Terme, Italy

## Background

Little is known about cardiac involvement in thalassemia intermedia (TI) using cardiovascular magnetic resonance (CMR). We investigated myocardial iron overload (MIO), biventricular parameters, and myocardial fibrosis in a large cohort of TI patients, underlying the differences between transfusion-dependent and non-transfusion-dependent patients.

## Methods

We studied 252 adult TI patients (119 females, 39.5 ± 10.4 years) enrolled in the MIOT Network. MIO was assessed using a multislice multiecho T2* approach. Biventricular function parameters were quantified by cine sequences. Myocardial fibrosis was evaluated by late gadolinium enhancement acquisitions.

## Results

One-hundred and eighty-eight (74.6%) patients showed no MIO in any segment, 56 (22%) had an heterogeneous distribution (52 with global heart T2*≥20 ms), and 8 (0.3%) showed an homogeneous MIO. Left ventricular (LV) and right ventricular (RV) dilatations were present in 113 (45%) and in 49 (19%) patients, respectively. LV dysfunction was present in the 18.0% of the cases while RV dysfunction in the 3.63%. High LV mass indexes were present in 22 (8.7%) patients. Fifty-two/227 (22.9%) patients showed myocardial fibrosis. Myocardial fibrosis was associated to LV dysfunction (P = 0.001) and high mass indexes (P = 0.038). One-hundred and fourteen patients were non-transfusion dependent (transfusion requirements absent or sporadic) while 138 patients were transfusion-dependent (regular transfusions). The mean age at start of chronic transfusions was 11.8 ± 12.3 years. Table 1 shows the comparison between the two groups. Non-transfusion-dependent patients showed significantly higher global heart T2* values and MIO with a global heart T2* < 20 ms was detected in two of them (one requiring occasional blood transfusions and one non transfused). Biventricular end-diastolic volume index, stroke volume index, left ventricular (LV) mass index, and LV cardiac index were significantly higher in the non-transfusion dependent group.

**Table 1:** 

	Non-transfusion-dependent	Transfusion-dependent	P
Age (years)	39.9 ± 11.5	39.2 ± 9.4	0.922

Sex (M/F)	67/47	66/72	0.083

Global heart T2* (ms)	38.8 ± 6.7	35.5 ± 9.2	0.014

MIO pattern, N (%):			

No MIO	92 (80.7)	96 (69.6)	0.103

Heterogeneous MIO with global T2* ≥ 20 ms	20 (17.5)	32 (23.2)	

Heterogeneous MIO with global T2* < 20 ms	1 (0.9)	3 (2.2)	

Homogeneous MIO	1 (0.9)	7 (5.1)	

LV end-diastolic volume index (ml/m2)	99.4 ± 19.6	92.9 ± 19.1	0.009

LV end-systolic volume index (ml/m2)	36.6 ± 11.4	34.9 ± 10.4	0.249

LV stroke volume index (ml/m2)	62.9 ± 12.4	58.6 ± 13.1	0.007

LV mass index (g/m2)	69.9 ± 13.9	63.9 ± 12.9	0.004

LV ejection fraction (%)	63.7 ± 6.8	62.5 ± 6.6	0.163

LV cardiac index (L/min/m2)	7.6 ± 2.3	6.5 ± 2.2	0.002

LGE, N (%)	20/105 (19)	32/122 (26.2)	0.199

RV end-diastolicvolume index (ml/m2)	92.0 ± 23.3	86.5 ± 20.8	0.048

RV end-systolic volume index (ml/m2)	32.7 ± 14.9	31.8 ± 11.3	0.571

RV stroke volume index (ml/m2)	58.5 ± 14.9	54.5 ± 14.3	0.017

RV ejection fraction (%)	64.7 ± 8.3	63.3 ± 7.5	0.168

MIO = myocardial iron overload; LV = left ventricular; RV = right ventricular

## Conclusions

CMR plays a key role in the management of TI patients. Heart iron (global heart T2* < 20 ms) was not common, but a quarter of the patients had some pathological segments. A consistent number of patients had the stigmata of the high cardiac output state cardiomyopathy. Myocardial fibrosis was related to the high cardiac output state. The signs of the high output state were controlled in the transfusion-dependent-patients.

## Funding

The MIOT project receives "no-profit support" from industrial sponsorships (Chiesi Farmaceutici S.p.A. and ApoPharma Inc.). This study was also supported by: "Ministero della Salute, fondi ex art. 12 D.Lgs. 502/92 e s.m.i., ricerca sanitaria finalizzata anno 2006" and "Fondazione L. Giambrone".

